# Predictive factors of anxiety and depression in COVID‐19 survivors: A cross‐sectional study

**DOI:** 10.1002/hsr2.1712

**Published:** 2023-11-21

**Authors:** Malihe Sohrabivafa, Roya Sadeghi, Forough Riahi, Abbas Rahimi Foroushani, Shirin Shahbazi Sighaldeh, Javad Zarei

**Affiliations:** ^1^ School of Public Health Tehran University of Medical Sciences Tehran Iran; ^2^ Department of Health Education and Promotion, School of Public Health Tehran University of Medical Sciences Tehran Iran; ^3^ Department of Psychiatry Ahvaz Jundishapur University of Medical Sciences Ahvaz Iran; ^4^ Department of Epidemiology and Biostatistics, School of Public Health Tehran University of Medical Sciences Tehran Iran; ^5^ Department of Reproductive Health, School of Nursing and Midwifery Tehran University of Medical Sciences Tehran Iran; ^6^ Department of Health Information Technology, School of Allied Medical Sciences Ahvaz Jundishapur University of Medical Sciences Ahvaz Iran

**Keywords:** anxiety, COVID‐19, depression, Iran, psychological intervention, survivors

## Abstract

**Background:**

Depression and anxiety are among the most critically recognized psychological complication of the COVID‐19 pandemic.

**Aim:**

This study aimed to examine the two predictors among COVID‐19 survivors in Ahvaz, Iran, in 2022.

**Methodology:**

Using a descriptive‐analytical design, 347 COVID‐19 survivors referred to hospitals in Ahvaz were meticulously examined. The database of the COVID‐19 registration system contained primary information about the samples. For data collection, questionnaires, including the Beck Anxiety and Depression Inventory and a demographic questionnaire, were utilized.

**Results:**

The results revealed a significant relationship between anxiety score and family size (*p* = 0.019), education level (*p* < 0.001), occupation (*p* = 0.015), household income status (*p* = 0.017), disease history (*p* = 0.017), ethnicity (*p* < 0.001), disease severity (*p* < 0.001), and quarantine period (*p* < 0.001). Furthermore, a significant correlation was observed between the average depression score and gender (*p* = 0.023), number of family households (*p* = 0.009), level of education (*p* < 0.001), occupation (*p* < 0.001), household income (*p* < 0.001), disease severity (*p* < 0.001), disease history (*p* < 0.001), and quarantine period (*p* < 0.001). Moreover, linear regression analysis indicated only a significant relationship between depression scores and the quarantine period variable (*p* < 0.001), among others. The simultaneous examination of all variables on depression disorder highlighted a meaningful relationship between depression score and disease history (*p* = 0.013), occupation (*p* = 0.002), household income status (*p* = 0.002), and family size (*p* = 0.039).

**Conclusions:**

This study revealed a significant relationship between certain demographic variables, such as quarantine period, disease history, employment status, household income status, and the number of family households, and an increase in the average depression and anxiety score.

## 1 INTRODUCTION

Coronavirus disease 2019 (COVID‐19), a highly contagious pandemic, has caused global public health concerns, including physical injuries and mental and emotional traumas. In addition to the 6.8 million deaths (as confirmed by the World Health Organization [WHO] as of March 3, 2023),^1^ it has also caused several psychological diseases, such as anxiety, stress, fear, hypochondriasis, obsessive‐compulsive disorder, posttraumatic stress disorder, corona phobia, sleeping disorder, depression, and social isolation.^2^, ^3^ COVID‐19 has produced numerous debilitating sociopsychological effects despite all successful health policies and concerted efforts to combat the virus.^4^ Disease and death fear, the spread of fake news and rumors, interference with daily life activities, travel restrictions, occupational and financial problems, and a reduction in social relations with coworkers, friends, and family are examples of several of the numerous negative effects of COVID‐19 that threaten human societies.^5^, ^6^ Similar psychological effects, including panic, depression, anxiety, and stress symptoms, have been reported in various countries due to the pandemic among ordinary people and health system workers.^7^ There is evidence that the high rate of infectiousness and unpredictability of the disease have caused widespread anxiety, cognitive ambiguity, and a decline in people's perception of safety.^8^

Depression is a serious and sometimes long‐lasting illness that affects many facets of a person's life.^9^ Depression is among the most severe and prevalent psychological disorders and a leading cause of disability in many countries. It burdens the health system heavily, accelerating the risk of premature death and significantly diminishing life quality.^10^ Numerous studies have focused solely on anxiety and depression levels during the pandemic, confirming the worldwide upward trend.^11^, ^12^, ^13^, ^14^, ^15^ Depression and anxiety have been reported as prevalent psychological complications among 30.1% and 33.4% of Iranian citizens, respectively, during the pandemic.^16^, ^17^ During the pandemic, a second study of 1498 individuals found that 36.6% of participants experienced stress, 57.9% anxiety, and 47.9% depression.^18^ The occurrence and prevalence of depression, anxiety, and stress are influenced by demographic characteristics, such as age, gender, income level, and urban living, according to studies.^19^, ^20^

In light of the above background, it appears imperative that public health policies, including both individual and collective psychological health, be implemented in the aftermath of the pandemic. Moreover, managing depression and anxiety in COVID‐19 survivors in Iran has also been understudied. Consequently, the current study aimed to investigate the predictors of anxiety and depression in COVID‐19 survivors in Ahvaz, Iran.

## 2 METHODS

This study was done based on the Strengthening the Reporting of Observational Studies in Epidemiology (STROBE) checklist for cross‐sectional studies.^21^

Covid survivors detained in the hospitals affiliated with Ahvaz Jundishapur University of Medical Sciences in the first half of 2021 constitute the participants in the study. The hospitals' databases of the COVID‐19 registration system including demographic data, admission data, the treatment procedures of the disease, signs, and symptoms (such as fever, cough, etc.), computerized tomography (CT)‐scans, polymerase chain reaction (PCR) tests, vital signs, pregnancy and background diseases, intensive care units (ICU) hospitalization, tracheal intubation, and the discharge status (discharge, dispatch, or death) of patients and suspected Covid patients were used in the study. Baloch et al.^12^ were modeled considering the number of samples to be statistically significant with 95% confidence and 80% test power if each correlation coefficient between variables is 0.15 or more. The following formulas were then used to calculate the size of the sample, and accordingly, 347 COVID survivors were selected from the list using a systematic random sampling method. A reserve list of 70 people was also made ready at hand according to the 20% drop rate of the sample size to be involved based on the entry criteria if the selected participants withdraw from the study.

$$W=\frac{1}{2}\log\left(\frac{1+0.15}{1-0.15}\right)=0.151,n=\frac{{{(Z}_{\alpha }+Z_{\beta })}^{2}}{W^{2}}+3=\frac{{\left(1.96+0.84\right)}^{2}}{0.151^{2}}+3=347.$$

Use was made of a random number generation software in the selection procedure from number 1 to 16,542 (the number of people registered in the COVID‐19 databank) in proportionate to the sample size for conducting a quantitative study. In so doing, a comprehensive list of patient files in the databank of the COVID‐19 registration system of Jundishapur University of Medical Sciences was prepared. The files were then numbered from 1 to 16542. According to the sample size (347 people), the *K* value was calculated as $k=\frac{16542}{347}=47/67\approx 47$, and a random number between 1 and 47 were selected (number 39 was selected randomly). The value of *k* = 47 was added to the number 39, and this process continued until the completion of 347 samples.

Inclusion criteria consisted of the 18–65 age range, the ability to speak, read and write in Persian, access to a smartphone and having informed consent. Exclusion criteria consisted of suffering or history of psychological disorders, taking psychiatric drugs, unwillingness or refusal to participate in the study, and incomplete completion of questionnaires.

All 347 selected people were contacted by phone and given explanations about the study objectives. The links to Beck Anxiety and Depression Inventories, demographics, and background questionnaires were sent to the willing participants through social media platforms. The scores obtained through the anxiety and depression inventories were used to categorize the participants into three groups, namely mild, moderate, and severe. Participants in the severe category were referred to a psychiatrist for treatment.

A researcher‐made demographic questionnaire including 12 demographic and clinical questions regarding the participants' particulars (age, gender, marital status, education level, occupation, household income status, ethnicity, place of residence, family size, quarantine status, other disease history, and COVID‐19 severity) was distributed at an early stage of data collection. Participants were then asked to fill out the Beck anxiety assessment inventory which includes 21 questions—each question covering four possible choices ranging from never, mild, moderate, to severe.

The four choices in each question of the Beck Anxiety Inventory are scored from 0 to 3. Each part of the test describes one of the common symptoms of anxiety (mental, physical, and fear symptoms), and the total score ranges from 0 to 63. The anxiety degree based on Beck Anxiety Inventory is determined in ranges 0–7 as none or minimal, 8–15 as mild, 16–25 as moderate, and 26–63 as severe. Much in the same vein, the Beck Depression Inventory includes 21 items for each of which four possible choices are provided to indicate the participant's feelings and behavior. The following scores are used to indicate the depression severity level: 0–13 (none or minimal depression), 14–19 (mild depression), 20–28 (moderate depression), and 29–63 (severe depression).

In measuring the reliability of the Beck Depression Inventory, a high‐level analysis of various attempts for internal consistency determination illustrated that the obtained coefficients ranged from 0.73 to 0.92 with an average of 0.86.^22^ The reliability of this study's questionnaire in a sample of 94 participants in Iran was as follows: Cronbach's *⍺* coefficient of 0.91%, the correlation coefficient between the two halves of the test of 0.89%, and the retest coefficient of 0.94%.^23^

Sharifi Daramadi and Ghasemi Davari estimated the reliability and validity of the Beck Depression Inventory as 0.85 and 0.76, respectively.^24^ Also, the correlation of the Beck Depression Inventory with its first edition was 0.93.^25^

The convergent validity of the Beck Depression Inventory was obtained through its simultaneous implementation with Beck Hopelessness Scale, Suicidal Thoughts Scale, and Beck Anxiety Inventory as 0.68, 0.37, and 0.60, respectively.^26^ Also, the correlation coefficient of the Beck Depression Inventory with the Hamilton psychiatric rating scale for depression is 0.73 and with the MMPI (Minnesota Multiphasic Personality Inventory) is 0.74.^24^ Beck Anxiety Inventory bears high validity according to the investigations of validity and reliability. The internal consistency coefficient of the anxiety scale inventory (alpha coefficient) and its reliability are reported as 0.92 and 0.75 with a 1‐week interval. Also, the anxiety scale inventory was obtained with a high internal consistency with correlation values in the range of 30–70. A high level of correlation (0.75) was obtained through the implementation of this test on 83 patients with a 1‐week interval for the retest.^27^ The correlation for depression inventory results was measured with Hamilton Rating Scale for Depression and Hamilton Anxiety Rating Scale as 0.51 and 0.25, respectively. In addition, the correlation between depression inventory results with depression inventory was 0.48.^28^

### 2.1 Statistical analysis

This study used the Kolmogorov–Smirnov test to investigate the normality of data. In the case of data normality, parametric tests of one‐way analysis of variance, *t* test, and in the case of nonnormality, the nonparametric tests of Kruskal–Wallis and Mann–Whitney were used. Multiple linear regression was used to simultaneously examine all variables on anxiety and depression,^29^ and all the statistical tests were performed by SPSS (version 24).

## 3 RESULTS

This study included 347 participants with an average age of 40.84 ± 10.15 years. Demographic characteristics are shown in Table 1. Background disease was observed among 274 participants (79%). According to the participants' report, 228 participants (66%) were quarantined at home when they were infected with COVID, and the last time of quarantine was reported between 1 and 7 days (Table 1).

**Table 1:** Descriptive analysis of participants' demographic characteristics (n = 347).

Variable		Number (%)	Variable		Number (%)
Age (years)	18–33	85 (24.5)	Occupation	Housewife	97 (28.0)
	34–47	174 (50.1)		Employee/nonemployee	214 (61.7)
	48 and upper	88 (25.4)		Other	36 (10.4)
Gender	Male	113 (32.6)	Residence status	Urban	335 (96.5)
	Female	234 (67.4)		Rural	12 (3.5)
Ethnicity	Fars	184 (27.1)	Education level	Under high school diploma	41 (11.8)
	Lor/Bakhtiari	94 (27.1)		High school diploma	77 (22.2)
	Arab	49 (14.1)		Academic degree	229 (66.0)
	Other	20 (5.8)	Other disease history	No	274 (79.0)
Quarantine period (day)	0	119 (34.3)		Yes	73 (21.0)
	1–7	56 (16.1)	COVID‐19 severity	Hospitalization	119 (34.3)
	8–14	112 (32.3)		Quarantine at home	228 (65.7)
	15 and upper	60 (17.3)	Household income	Sufficient for life	148 (42.7)
Family size	1–3	169 (48.7)		Sufficient for life to some extent	153 (44.1)
	4 and upper	178 (51.3)		Not sufficient for life	46 (13.3)
Marital status	Single	54 (15.6)			
	Married	275 (79.3)			
	Divorced/deceased spouse	18 (5.2)			

Analysis of the frequency of anxiety among participants by gender indicates that 11 (33.1%) and 74 (21.3%) participants have no or the minimum and the most severe depression, respectively. In addition, the results reveal that the anxiety degree (mild to severe) is more in women than in men (Table 2). Meanwhile, the frequency distribution of the depression degree among the participants by gender demonstrates that 247 participants (71.2%) have the lowest depression degree and 21 participants (6.1%) have a severe depression degree, among which the depression degree (mild to severe) is reported more in women than in men (Table 2). Considering the relationship between the demographic factors under study and anxiety disorder among the participants, there is a significant relationship between depression average score and the variables of the family size (*p* = 0.019), education level (*p* < 0.001), ethnicity (*p* < 0.001), occupation (*p* = 0.015), family income status (*p* = 0.017), other disease history (*p* = 0.017), COVID‐19 severity (*p* < 0.001), and quarantine period (*p* < 0.001). Meanwhile, there is not a significant relationship between anxiety and other variables, including gender (*p* = 0.530), residence (*p* = 0.465), marital status (*p* = 0.208), and age (*p* = 0.319) (Table 3). According to the results, the average anxiety score in families with four or more households is the highest (17.69 ± 13.77). Analysis of the relationship between education level and average anxiety score indicates that there is the highest average anxiety score among the participants with lower than high school education (24.43 ± 16.02). Also, Arabs, among other ethnicities, experience the highest anxiety level (22.34 ± 14.51). Analysis of the occupation factor with anxiety scores shows that the highest and lowest average anxiety score belongs to the household group (19.41 ± 14.43) and employee/nonemployee occupational group (14.44 ± 12.07), respectively. The highest (18.32 ± 13.20) and lowest (62 ± 11.44) average anxiety scores are observed for participants without and with sufficient income for subsistence, respectively. The average anxiety score is higher in participants with other disease histories (19.39 ± 14.37) than in participants without another disease history (15.17 ± 12.70). In addition, the average anxiety score of participants who were hospitalized (22.70 ± 15.70) is higher than that of the participants quarantined at home (12.60 ± 10.04). Eventually, the lowest average anxiety score relates to the participants quarantined at home between 1 and 7 days (9.44 ± 10.03). Furthermore, there is a significant relationship between the average depression and gender (*p* = 0.023), family size (*p* = 0.009), education level (*p* < 0.001), occupation (*p* < 0.001), household income (*p* < 0.001), other disease history (*p* < 0.001), COVID‐19 severity (*p* < 0.001), and the quarantine period (*p* < 0.001). On the other hand, there is no significant relationship between depression and other variables such as ethnicity (*p* = 0.098), residence (*p* = 0.392), marital status (*p* = 0.200), and age (*p* = 0.821), (Table 3). The analysis also reveals that the average score of anxiety and depression is higher in participants with family size four or more than those living in a family of 1–3 households. Regarding the education level, the highest level of anxiety score is observed in participants with lower than high school education. Moreover, Arabs experience higher anxiety and depression compared with the other ethnic groups in the study. Considering the relationship between occupation and anxiety score, it is observed that the housewife group and the employee/nonemployee group experience the highest and the lowest average anxiety and depression, respectively. Also, the highest and the lowest average anxiety scores are observed for the participants without and with sufficient income, respectively. Furthermore, the average anxiety score is higher in participants with a disease history than in participants without it. Finally, the average anxiety score of participants hospitalized is higher than that of the participants quarantined at home. In the same vein, the lowest average anxiety score is reported for the participants quarantined at home between 1 and 7 days. Multiple linear regression was used for the simultaneous examination of the variables on anxiety and depression disorders. The results of multiple linear regression demonstrate that the relationship between anxiety scores is significant only with the quarantine period variable (*p* < 0.001). In this regard, the regression coefficient for the anxiety score of the participants quarantined at home is equal to −7.373, with a standard error of 1.609. In other words, the average anxiety score is 7.373 points less in the quarantined participants in comparison with those who were not quarantined (i.e., the hospitalized participants), which is statistically significant (*p* < 0.001) (Table 4). Furthermore, in the simultaneous analyses of all variables on depression disorder, it was revealed that among the significant variables from the previous stage, there is a significant relationship between the depression score and other disease history (*p* = 0.013), occupation (*p* = 0.002), household income (*p* = 0.002), and family size (*p* = 0.039) (Table 4).

**Table 2:** Descriptive analysis of anxiety and depression in participants by gender.

Degree	Anxiety		Depression	
	Male number (%)	Female number (%)	Male number (%)	Female number (%)
None or minimal	44 (38.9)	71 (30.3)	88 (77.9)	159 (67.9)
Mild	19 (16.8)	64 (27.4)	11 (9.7)	30 (12.8)
Medium	29 (25.7)	46 (19.7)	9 (8.0)	29 (12.4)
Sever	21 (18.6)	53 (22.6)	5 (4.4)	16 (6.8)
Total sum	113 (100.0)	234 (100.0)	113 (100.0)	234 (100.0)

**Table 3:** Analysis of anxiety and depression scores by demographic variables.

	Variable	Anxiety standard deviation ± average	*p* Value	Depression standard deviation ± average	*p* Value*
Age (years)	18–33	14.37 ± 11.47	0.319	9.07 ± 10.85	0.821
	34–47	17.08 ± 13.64		9.25 ± 10.38	
	48 and upper	15.68 ± 13.67		10.68 ± 12.42	
Gender	Male	15.63 ± 13.29	0.530	7.74 ± 10.26	0.023
	Female	16.27 ± 13.12		10.45 ± 11.30	
Family size	1–3	14.34 ± 12.30	0.019	8.01 ± 10.23	0.009
	4 and upper	17.69 ± 13.77		11.04 ± 11.58	
Marital status	Single	13.68 ± 9.79	0.208	8.16 ± 8.90	0.200
	Married	16.16 ± 13.49		9.63 ± 11.41	
	Divorced/deceased spouse	21.72 ± 15.58		12.83 ± 10.63	
Education level	Under high school diploma	24.43 ± 16.02	<0.001	14.58 ± 13.38	<0.001
	High school diploma	19.05 ± 15.01		12.74 ± 11.21	
	Academic degree	13.56 ± 10.99		7.60 ± 9.98	
Residence status	Urban	15.98 ± 13.17	0.465	9.48 ± 11.01	0.392
	Rural	18.33 ± 13.35		12.00 ± 11.90	
Ethnicity	Fars	12.88 ± 11.02	<0.001	8.36 ± 9.86	0.098
	Lor/Bakhtiari	18.38 ± 14.29		9.82 ± 11.52	
	Arab	22.34 ± 14.51		12.42 ± 12.66	
	Other	19.41 ± 14.43		13.16 ± 11.90	
Occupation	Housewife	19.41 ± 14.43	0.015	13.16 ± 11.90	<0.001
	Employee/nonemployee	14.44 ± 12.07		7.22 ± 9.24	
	Other	16.69 ± 14.36		13.80 ± 14.40	
Household income	Sufficient for life	18.32 ± 13.20	0.017	17.89 ± 13.74	<0.001
	Sufficient for life to some extent	13.62 ± 11.44		6.79 ± 8.58	
	Not sufficient for life	17.74 ± 14.36		9.75 ± 11.02	
Other disease history	No	15.17 ± 12.70	0.017	8.31 ± 10.22	<0.001
	Yes	19.39 ± 14.37		14.28 ± 12.66	
COVID‐19 severity	Quarantine at home	12.60 ± 10.04	<0.001	8.12 ± 10.36	<0.001
	Hospitalization	22.70 ± 15.70		12.33 ± 11.78	
Quarantine period (day)	0	22.81 ± 15.70	<0.001	12.42 ± 11.77	<0.001
	1–7	9.44 ± 10.03		7.48 ± 11.04	
	8–14	12.85 ± 9.95		7.36 ± 8.94	
	15 and upper	15.01 ± 9.60		10.06 ± 11.99	

* *p* Value results from Kruskal–Wallis and Mann–Whitney tests.

**Table 4:** Linear regression analysis to examine the relationship between anxiety and depression.

Variable	Anxiety				Variable	Depression			
	*β*	Standard deviation	*p* Value	Confidence interval		*β*	Standard deviation	*p* Value	Confidence interval
Family size	2.066	1.313	0.117	(−0.518,4.649)	Gender	1.042	1.307	0.426	(−1.528,3.612)
Education level	−2.577	1.650	0.119	(−5.823,0.669)	Other disease history	3.554	1.415	0.013	(0.770,6.338)
Ethnicity	2.654	1.461	0.070	(−0.221,5.528)	Education level	−1.309	1.404	0.352	(−4.070,1.452)
Occupation	−1.457	1.455	0.317	(−4.319,1.405)	Job	−4.109	1.345	0.002	(−6.754, −1.463)
Household income	−2.222	1.408	0.116	(−4.993,0.548)	Household income	−3.672	1.174	0.002	(−5.981, −1.362)
Other disease history	0.506	1.659	0.761	(−2.757,3.769)	Family size	2.309	1.112	0.039	(0.122,4.497)
Quarantine period	−7.373	1.609	<0.001	(−10.537, −4.209)	Quarantine period	−1.917	1.280	0.135	(−4.435,0.601)

## 4 DISCUSSION

Psychological complications are one of the most severe consequences of the COVID‐19 pandemic, alongside social and economic damages and high mortality. In addition to increased depression, stress, anxiety, and fear among various social groups, it may also result in unhealthy conditions, chronic diseases, and risky behaviors, such as increased smoking, drug abuse, alcohol consumption, aggressive behaviors, and suicidal thoughts.^14^ The psychological effects of the virus will be felt long after the pandemic, and the confrontation of the mind and psyche with this phenomenon will continue to influence the mentalities, thoughts, emotions, and life processes of the affected individuals, families, and society for years.^30^ Consequently, the present study aimed to investigate the demographic and background variables associated with anxiety and depression in COVID‐19 survivors.

The results generally reveal a significant relationship between quarantine period and anxiety, while a significant relationship exists between depression and disease history, occupation, household income status, and family size. In this regard, the regression coefficient indicates that the average anxiety score of participants quarantined at home is 7.373 points lower than that of participants hospitalized. After surviving the pandemic, patients quarantined at home despite fears of disease transmission to other family households are less likely to develop anxiety‐related psychological disorders. Nonetheless, it is demonstrated that there is a positive correlation between the quarantine period and the average anxiety and depression scores. Thus, it emphasizes the significance of social isolation caused by quarantine concerning the increased likelihood of anxiety and depressive disorders, as suggested by other studies that anxiety and depression symptoms can remain after recovery from COVID‐19.^31^, ^32^ On the other hand, regression coefficient results indicate that the average depression score is lower among patients with sufficient income levels than among patients who consider their household income somewhat or entirely insufficient. The average depression score is higher in families with more than four members than in families with one to three. In addition, examining the relationship between demographic variables and average depression score reveals that the average depression score of participants with a disease history is 3.554% higher than that of participants without a disease history. In addition, the average anxiety score among employees and nonemployees is 4.109 points lower than that of homemakers and other groups.

Studies indicate that COVID‐19 has caused psychological disorders and mental health issues, such as anxiety and depression, in various societies.^11^ For example, a longitudinal study revealed that more than half of hospitalized COVID‐19 patients experienced COVID‐19 syndrome and disease symptoms between 3 and 6 months after discharge.^33^ Another study investigated the long‐term symptoms of depression and anxiety in Spanish patients who survived COVID‐19; in the study, 1969 men and women hospitalized for 6–8 months were examined. The prevalence of anxiety and depression in COVID‐19 survivors was 16.2% and 19.7%, respectively.^34^ Consistent with these studies, the present study found that 66.8% of COVID‐19 survivors suffered from mild to severe anxiety disorder after recovery.

Nasirzadeh et al.^13^ studied stress, anxiety, depression, and resilience in 453 Rafsanjan (Iran) households during the pandemic. They observed a significant trend of psychological disorders such as depression, anxiety, and stress increasing over time. In addition, they found a significant correlation between anxiety level and education level, occupation, and age, as well as between depression level and occupation.^13^

The present study's linear regression analysis reveals a significant correlation between ethnicity, quarantine period, and anxiety score. In addition, there is a significant correlation between the depression score and disease history, occupation, household income, and the number of family households. Considering employment status, the results indicate that the unemployed, university students, and retirees have higher depression scores than the other participants. Consequently, the effect of social isolation caused by unemployment and insufficient income during the pandemic is emphasized. Contrary to Othman and Baloch et al., our findings suggest an inverse relationship between the average anxiety and depression scores and education level, meaning that the higher the education level, the lower the average anxiety and depression scores.^12^, ^35^

According to a study conducted in the southern regions of Sistan and Baluchistan (Iran), gender, employment, and insurance coverage variables have a statistically significant relationship with anxiety and depression caused by the pandemic.^12^

A recent study revealed that men score higher on average for anxiety and depression than women.^12^ However, the current study shows no statistically significant differences between the average anxiety scores of men and women, whereas the average depression scores of men are higher (*p* = 0.023). Some epidemiological studies have reported that women are more likely to suffer from depressive disorders than men. However, the present study's findings contradict these findings.^36^

Arya Puran et al. examined the prevalence of depression, anxiety, and suicidal ideation among nurses during the COVID‐19 outbreak and their relationship to demographic variables. They observed that depression is more prevalent among female nurses than male nurses.^14^ In a study conducted in Wuhan, China, on the correlation between gender and stress and anxiety disorders during the COVID‐19 pandemic, female nurses exhibited more stress and anxiety symptoms when working with COVID‐19 patients.^11^ In another study on the influence of sociodemographic factors on anxiety and depression levels among university students and faculty households at Jouf University during the pandemic, it was observed that women suffer from moderate anxiety and depression more than men.^37^

A study examining the occurrence and outcomes of psychological interventions during the pandemic found that prolonged quarantine duration, concern about the spread of the disease to others, frustration and fatigue, and a lack of information about the disease and its symptoms can contribute to the development of psychological disorders.^15^ Another study observed that the education level of nurses influences their depression rate. The marital status and workplace of nurses also play a significant role in anxiety rates. Findings indicated that B.Sc. degree holders had more suicidal thoughts than M.Sc. holders. In addition, single nurses had higher anxiety and suicidal ideation rates than their married counterparts. Finally, it was observed that emergency department, CCU, and ICU nurses had the highest average anxiety rate.^14^ However, according to the results of the current study, there is no correlation between the average anxiety and depression scores and marital status and age variables. An analysis of the results revealed that the average anxiety and depression scores are higher among divorced or widowed individuals. Similarly, Ramandi et al. found a higher incidence of depression and anxiety among widows.^38^

In addition, anxiety and depression are more prevalent among those aged 34–47 and 48 years and older, respectively. Similarly, Abadi et al. found that the rate of depression is higher among those aged 31–49, which may be due to a lack of energy, a busy lifestyle, and diminished social interaction.^39^ However, a separate study found that the likelihood of suffering from an anxiety disorder increases with age between 30 and 39.^40^ According to a study on the relationship between demographic variables and the level of anxiety and stress among students and faculty households of Jouf University during the COVID‐19 pandemic using the Beck Inventory, Ganji et al. found that ethnicity and age are among the most influential factors in anxiety and depression levels, such that the average anxiety and depression rates were higher among individuals aged 20–30 and of Saudi Arabian nationality.^37^ The present study found a significant correlation between ethnicity and the average anxiety rate but none between ethnicity and the average depression rate. Table 3 indicates that participants of Arab ethnicity experience the highest rates of depression and anxiety.

The inclusion of multiple demographic variables, ethnic diversity, selection of participants from a vast area, and timing of data collection relative to quarantine restrictions in Iran are particular strengths of the present study.

### 4.1 Study limitations

On the other hand, the current study has several drawbacks. First, data reliability is diminished due to self‐reporting and online questionnaire completion. Due to the lengthy number of questions, participants may not have been in a good mood while completing the questionnaires. Thirdly, this study was restricted to samples that were able to use the internet and participate in the study online, as well as those who have survived COVID‐19 in the hospitals of Ahvaz and whose records were available in the COVID‐19 registration system database. These particulars ultimately limit the generalizability of the findings. Conducting investigations on a larger scale with a larger sample size is recommended in future studies. In addition to investigating the demographic characteristics, the relationship between anxiety and depression disorders, coping styles, and environmental factors will be examined. In addition, future studies can improve the generalizability of the samples by including patients from other regions of the country.

## 5 CONCLUSION

The COVID‐19 pandemic has caused physical health concerns and significantly increased the incidence of adverse psychological complications, such as depression and anxiety, in infected individuals, with some survivors experiencing long‐term effects. Potentially, psychological capital interventions should be tailored to at‐risk populations with benefits for current and upcoming pandemics and health crises. In these unprecedented times, positive health‐related behaviors must be adopted or maintained through effective health promotion strategies to reduce the acute and chronic increase in psychological distress. Finally, public health messages activating self‐management cognitive resources, processes, and psychological capital can foster health‐protective behaviors. In addition, this study revealed a significant correlation between certain demographic variables, such as quarantine period, disease history, employment status, household income status, and the number of family households, and an increase in the average depression and anxiety score. Patients and survivors of COVID‐19 must therefore be screened for and treated for psychological disorders like depression and anxiety. To this end, it is suggested that vulnerable groups receive timely and appropriate psychological interventions through counseling and psychological services and increased financial and psychological support.

## AUTHOR CONTRIBUTIONS

**Malihe Sohrabivafa**: Data curation; formal analysis; resources; software; writing—original draft; writing—review and editing. **Roya Sadeghi**: Data curation; formal analysis; methodology; resources; visualization; writing—original draft; writing—review and editing. **Forough Riahi**: Conceptualization; supervision; writing—review and editing. **Abbas Rahimi Foroushani**: Methodology; software; validation; visualization; writing—review and editing. **Shirin Shahbazi Sighaldeh**: Resources; validation; writing—original draft; writing—review and editing. **Javad Zarei**: Data curation; formal analysis; resources; writing—review and editing.

## CONFLICT OF INTEREST STATEMENT

The authors declare no conflict of interest.

## ETHICS STATEMENT

This study was approved by the Tehran University of Medical Sciences with IR.TUMS.SPH.REC.1401.084 Ethics Code. In the present study, the explanation of the purpose, research nature and research methodology, voluntary participation in the study and obtaining written consent, and confidentiality of personal information were observed, and the participant's name in the questionnaire remained confidential.

## TRANSPARENCY STATEMENT

The lead author Roya Sadeghi affirms that this manuscript is an honest, accurate, and transparent account of the study being reported; that no important aspects of the study have been omitted; and that any discrepancies from the study as planned (and, if relevant, registered) have been explained.

## Data Availability

The data that support the findings of this study are available from the corresponding author upon reasonable request.

## References

[1] Cascella M , Rajnik M , Aleem A , Dulebohn SC , Di Napoli R . Features, Evaluation, and Treatment of Coronavirus (COVID‐19). StatPearls Publishing LLC; 2023. 32150360

[2] Shahyad S , Mohammadi MT . Psychological impacts of Covid‐19 outbreak on mental health status of society individuals: a narrative review. J Mil Med. 2020;22(2):184‐192.

[3] Samimi Ardestani SM , Khosravani V , Sharifi Bastan F , Baloğlu M . The Persian version of the COVID‐19 Phobia Scale (Persian‐C19P‐S) and the differences in COVID‐19‐related phobic reactions in patients with anxiety disorders. Int J Ment Health Addict. 2022;20(4):2419‐2435. 33841053 10.1007/s11469-021-00523-0 PMC8025735

[4] Barach P , Fisher SD , Adams MJ , et al. Disruption of healthcare: will the COVID pandemic worsen non‐COVID outcomes and disease outbreaks? Prog Pediatr Cardiol. 2020;59:101254. 32837144 10.1016/j.ppedcard.2020.101254 PMC7274978

[5] Saffarinia M . The prediction of mental health based on the anxiety and the social cohesion that caused by Coronavirus. Quart Social Psychol Res. 2020;9(36):129‐141.

[6] Lee SS , Shim Y , Choi J , Choi I . Paradoxical impacts of social relationship on well‐being during the COVID‐19 pandemic. J Happiness Stud. 2023;24(2):745‐767. 36686601 10.1007/s10902-022-00614-2 PMC9838380

[7] Eghbali A , Vahedi H , Rezaei R , Fathi A . The effectiveness of mindfulness‐based stress reduction training on depression, anxiety and stress in people at risk for COVID‐19. J Health Care. 2021;22(4):306‐317.

[8] Akhlaghifard M , Meraji N . Predicting corona anxiety based on emotional distress (depression, anxiety and stress) and spiritual health in nurses and aides. Rooyesh‐e‐Ravanshenasi J. 2021;10(6):161‐170.

[9] Baghiani Moghaddam M , Ehrampoush M , Rahimi B , Aminian A , Aram M . Prevalence of depression among successful and unsuccessful students of Public Health and Nursing‐Midwifery schools of Shahid Sadoughi University of Medical Sciences in 2008. J Med Educ Dev. 2012;6(1):17‐24.

[10] Musarezaie A , Momeni‐Ghalehghasemi T , Musarezaie N , Moeini M , Khodaee M . Investigate the Prevalence of Depression and its association with Demographic variables in employees. J Nurs Educ. 2014;2(3):37‐45.

[11] Lai J , Ma S , Wang Y , et al. Factors associated with mental health outcomes among health care workers exposed to coronavirus disease 2019. JAMA Netw Open. 2020;3(3):e203976. 32202646 10.1001/jamanetworkopen.2020.3976 PMC7090843

[12] Balouch V , Vatanparast M , Dadpisheh S , Mirkazehi Rigi Z . Stress, anxiety and depression status in the population of southern Sistan and Baluchestan province (coastal area) in the COVID‐19 epidemic in 2020. J Mar Med. 2021;2(4):226‐236.

[13] Nasirzadeh M , Akhondi M , Jamalizadeh nooq A , Khorramnia S . A survey on stress, anxiety, depression and resilience due to the prevalence of COVID‐19 among Anar City Households in 2020: a short report. J Rafsanjan Uni Med Sci. 2020;19(8):889‐898.

[14] Ariapooran S , Amirimanesh M . Depression, anxiety and suicidal ideation of nurses in the outbreak of COVID‐19: the role of demographic variables. J Arak Uni Med Sci. 2020;23(5):724‐739.

[15] Shahed hagh ghadam H , Fathi Ashtiani A , Rahnejat AM , et al. Psychological consequences and interventions during the COVID‐19 pandemic: narrative review. J Mar Med. 2020;2(1):1‐11.

[16] Maroufizadeh S , Pourshaikhian M , Pourramzani A , Sheikholeslami F , Moghadamnia MT , Alavi SA . Prevalence of anxiety and depression in the Iranian general population during the COVID‐19 pandemic: a web‐based cross‐sectional study. Iran J Psychiatry. 2022;17(2):230‐239. 36262765 10.21203/rs.3.rs-39082/v1 PMC9533345

[17] Babadi F , Bazmi A , Araban M . Association between the fear induced by the COVID‐19 and the level of depression, anxiety, and stress among dental students: a cross‐sectional study. Health Educ Health Promot. 2021;9(1):19‐24.

[18] Khademian F , Delavari S , Koohjani Z , Khademian Z . An investigation of depression, anxiety, and stress and its relating factors during COVID‐19 pandemic in Iran. BMC Public Health. 2021;21(1):275. 33535992 10.1186/s12889-021-10329-3 PMC7856614

[19] Robab BG , Zeynab K , Jalil B . Comparison of five big personality factors in depressive disorder patients and obsessive–compulsive disorders with normal individuals. Knowledge Res Appl Psychol. 2013;14(1):110‐117.

[20] Ebstrup JF , Eplov LF , Pisinger C , Jørgensen T . Association between the Five Factor personality traits and perceived stress: is the effect mediated by general self‐efficacy? Anxiety Stress Coping. 2011;24(4):407‐419. 21213153 10.1080/10615806.2010.540012

[21] von Elm E , Altman DG , Egger M , Pocock SJ , Gøtzsche PC , Vandenbroucke JP . The Strengthening the Reporting of Observational Studies in Epidemiology (STROBE) statement: guidelines for reporting observational studies. J Clin Epidemiol. 2008;61(4):344‐349. 18313558 10.1016/j.jclinepi.2007.11.008

[22] Groth‐Marnat G . Handbook of Psychological Assessment. 6th ed. Roshd; 2016.

[23] Fata L , Birashk B , Atefvahid M , Dabson K . Meaning assignment structures/schema, emotional states and cognitive processing of emotional information: comparing two conceptual frameworks. Iran. J Psychiatry Clin. Psychol. 2005;11(3):312‐326.

[24] Sharifi Daramadi P , Qassemi Davari L . Comparison of emotional intelligence, self‐esteem, and depression in crime victim and non‐victim girls aged between 15–18 in Tehran (2010–2011). Psychol Except Individuals. 2012;2(7):115‐132.

[25] Modares M . Effectiveness of group dialectical behavior therapy (based on core mindfulness, distress tolerance and emotion regulation components) on depressive symptoms in university students. J Fundamentals Mental Health. 2011;13(50):35‐124.

[26] Beck AT , Steer RA , Ball R , Ranieri WF . Comparison of Beck Depression Inventories‐IA and‐II in psychiatric outpatients. J Pers Assess. 1996;67(3):588‐597. 8991972 10.1207/s15327752jpa6703_13

[27] Salari‐Moghaddam S , Ranjbar AR , Fathi‐Ashtiani A . Validity and Reliability measurement of the Persian version of anxiety Control Questionnaire. J Clin Psychol. 2018;9(4):33‐43.

[28] Khodabakhsh Pirkalani R , Rahim Jamarouni H . Effectiveness of mixed cognitive‐behaviorial therapy and mindfulness based stressreduction in treating a case of generalized anxiety disorder. Clin Psychol Studies. 2013;4(13):121‐147.

[29] A A. Sampling Theory and Its Applications. 8 ed. University Publishing Center; 2015.

[30] Min W , Wang B , Li J , et al. The expression and significance of five types of miRNAs in breast cancer. Med Sci Monit Basic Res. 2014;20:97‐104. 25047098 10.12659/MSMBR.891246 PMC4117676

[31] Xiao C . A novel approach of consultation on 2019 novel coronavirus (COVID‐19)‐related psychological and mental problems: structured letter therapy. Psychiatry Investig. 2020;17(2):175‐176. 10.30773/pi.2020.0047 PMC7047000 32093461

[32] Avula VCR , Amalakanti S , Fasiha MS . Anxiety and depression in COVID survivors—a cross‐sectional study six months past the acute illness. Cureus. 2023;15(8):e42971. 37671223 10.7759/cureus.42971 PMC10475854

[33] D'Cruz RF , Waller MD , Perrin F , et al. Chest radiography is a poor predictor of respiratory symptoms and functional impairment in survivors of severe COVID‐19 pneumonia. ERJ Open Res. 2021;7(1):00655‐2020. 33575312 10.1183/23120541.00655-2020 PMC7585700

[34] Fernández‐de‐Las‐Peñas C , Pellicer‐Valero OJ , Navarro‐Pardo E , Rodríguez‐Jiménez J , Martín‐Guerrero JD , Cigarán‐Méndez M . The number of symptoms at the acute COVID‐19 phase is associated with anxiety and depressive long‐term post‐COVID symptoms: a multicenter study. J Psychosom Res. 2021;150:110625. 34563747 10.1016/j.jpsychores.2021.110625 PMC8455234

[35] Kamal NM , Othman N . Depression, anxiety, and stress in the time of COVID‐19 pandemic in Kurdistan Region, Iraq. Kurdistan J Appl Res. 2020;5:37‐44.

[36] Zhou SJ , Zhang LG , Wang LL , et al. Prevalence and socio‐demographic correlates of psychological health problems in Chinese adolescents during the outbreak of COVID‐19. Eur Child Adolesc Psychiatry. 2020;29(6):749‐758. 32363492 10.1007/s00787-020-01541-4 PMC7196181

[37] Ganji KK , Alam MK , Siddiqui AA , Munisekhar MS , Alduraywish A . COVID‐19 and stress: an evaluation using Beck's depression and anxiety inventory among college students and faculty members of Jouf University. Work. 2022;72:399‐407. 35527603 10.3233/WOR-210346

[38] Ramandi MMA , Yarmohammadi H , Beikmohammadi S , Fahimi BHH , Amirabadizadeh A . Factors associated with the psychological status during the Coronavirus pandemic, baseline data from an Iranian Province. Caspian J Intern Med. 2020;11(0):484‐494. 33425265 10.22088/cjim.11.0. PMC7780878

[39] Abadi TSH , Askari M , Miri K , Nia MN . Depression, stress and anxiety of nurses in COVID‐19 pandemic in Nohe‐Dey Hospital in Torbat‐e‐Heydariyeh city, Iran. J Mil Med. 2020;22(6):526‐533.

[40] Hassannia L , Taghizadeh F , Moosazadeh M , et al. Anxiety and depression in health workers and general population during COVID‐19 in Iran: a cross‐sectional study. Neuropsychopharmacol Rep. 2021;41(1):40‐49. 33369264 10.1002/npr2.12153 PMC8182959

